# “It’s a stressful, trying time for the caretaker”: an interpretive description qualitative study of postoperative transitions in care for older adults with frailty from the perspectives of informal caregivers

**DOI:** 10.1186/s12877-024-04826-4

**Published:** 2024-03-11

**Authors:** Emily Hladkowicz, Mohammad Auais, Gurlavine Kidd, Daniel I McIsaac, Jordan Miller

**Affiliations:** 1 School of Rehabilitation Therapy, Queen’s University, Kingston, Canada; 2 Clinical Epidemiology Program, Ottawa Hospital Research Institute, Ottawa, Canada; 3 Patient Partner, The Ottawa Hospital Research Institute, Ottawa, Canada; 4 Department of Anesthesiology and Pain Medicine, University of Ottawa, The Ottawa Hospital, Civic Campus Room B311, 1053 Carling Ave, Mail Stop 249, K1Y 4E9 Ottawa, ON Canada; 5 School of Epidemiology & Public Health, University of Ottawa, Ottawa, Canada

**Keywords:** Transitions in care, Informal caregivers, Surgery, Qualitative methods, Frailty

## Abstract

**Background:**

Older adults with frailty have surgery at a high rate. Informal caregivers often support the postoperative transition in care. Despite the growing need for family and caregiver support for this population, little is known about the experience of providing informal care to older adults with frailty during the postoperative transition in care. The purpose of this study was to explore what is important during a postoperative transition in care for older adults with frailty from the perspective of informal caregivers.

**Methods:**

This was a qualitative study using an interpretive description methodology. Seven informal caregivers to older adults [aged ≥ 65 years with frailty (Clinical Frailty Scale score ≥ 4) who had an inpatient elective surgery] participated in a telephone-based, semi-structured interview. Audio files were transcribed and analyzed using reflexive thematic analysis.

**Results:**

Four themes were constructed: (1) being informed about what to expect after surgery; (2) accessible communication with care providers; (3) homecare resources are needed for the patient; and (4) a support network for the caregivers. Theme 4 included two sub-themes: (a) respite and emotional support and (b) occupational support.

**Conclusions:**

Transitions in care present challenges for informal caregivers of older adults with frailty, who play an important role in successful transitions. Future postoperative transitional care programs should consider making targeted information, accessible communication, and support networks available for caregivers as part of facilitating successful transitions in care.

**Supplementary Information:**

The online version contains supplementary material available at 10.1186/s12877-024-04826-4.

## Introduction

The majority of care received by older adults is from informal caregivers [1, 2]. While the definition of an informal caregiver ranges from an adult child or a co-residing spouse providing care as needed, to someone who provides ongoing support with Activities of Daily Living (ADLs), the defining feature is that an informal caregiver provides some type of unpaid support [3].Typically, informal caregivers have a social relationship with the person they are providing care for, and could include a spouse, child, other relative, neighbour or friend [4].

Having support from an informal caregiver following a hospital stay is associated with a decrease in healthcare utilization, including shorter hospital length of stay, reduced homecare services and a lower likelihood of transitioning into long-term care [4]. The transition out of hospital is a challenging time that can lead to poor quality of care for older adults [5]. Transitional care is defined as, “a set of actions designed to ensure the coordination and continuity of healthcare as patients transfer between different locations or different levels of care within the same location” [6]. Older adults having surgery often require the support of informal caregivers during the postoperative transition in care [7], so ensuring quality transitional care is crucial for older patients and their caregivers [6]. Importantly, older adults who live at home with family are more likely to be discharged home after surgery than older adults who live alone [7].

Older surgical patients strongly prioritize going home after surgery [8], however, research shows that older adults develop the same loss of independence after surgery regardless of living with family or alone [7]. This results in challenges and negative impacts on well-being for informal caregivers due to their vital roles in supporting the older adult who has transitioned home. Evidence indicates that caregiver strain is increased after surgery compared to pre-surgery, peaking at discharge and in the 2 weeks after surgery [9]. The prevalence of caregiver strain is likely to increase, as patient-related factors associated with increased caregiver strain (e.g., older age, physical or cognitive impairment, comorbidities) are increasingly common among surgical patients [9]. Frailty, a state of vulnerability to adverse health outcomes related to accumulation of multidimensional deficits, is highly prevalent among older surgical patients (∼ 40%), and is associated with a two-fold increase in patient-reported disability after surgery [10, 11]. Therefore, understanding the experiences and priorities of individuals providing informal postoperative care to older adults with frailty is important to support the integral role that such caregivers play in the postoperative transition in care, and in planning postoperative transitional care interventions, healthcare planning, and health system policies. Accordingly, the purpose of this study was to explore what is important to informal caregivers during a postoperative transition in care for older adults with frailty.

## Materials & methods

### Study design

This was a qualitative study conducted in the interpretive description tradition [12]. The purpose of interpretive description is to create knowledge that can be applied in a clinical, real-world setting [12]. Interpretive description is an applicable methodology to address the current research question as new knowledge is required to support informal caregivers during postoperative transitions in care. Semi-structured interviews were conducted to explore what is important to informal caregivers as they help support a postoperative transition in care for older adults with frailty. Interpretive description emphasizes that knowledge does not stay the same as it evolves and changes over time [12]. As such, this research is positioned within a constructivist paradigm, where multiple realities and perspectives exist, eliminating the possibility of one, single truth [13]. Informal caregivers were recruited in dyads with older adults with frailty who had inpatient elective surgery. The results of the patient interviews have been previously published [14].

### Reflexivity statement

EH is a female Doctoral student in Aging & Health and a Clinical Research Associate in a perioperative medicine research program. EH has completed a frailty fellowship through the Canadian Frailty Network and was an informal, essential caregiver to her grandfather in long-term care. MA is an Assistant Professor in the School of Rehabilitation Therapy and is a registered physical therapist with expertise in geriatric rehabilitation. GK is a patient partner with lived experience as an older adult who has had inpatient elective surgery and is a retired Social Worker. DIM is an anesthesiologist and scientist who conducts clinical trials to improve patient and system-level outcomes of older people having surgery. JM is an Assistant Professor in the School of Rehabilitation and conducts quantitative and qualitative research in the areas of health services, pain management and primary care.

### Setting and participants

Ethics approval was obtained from The Ottawa Health Sciences Network– Research Ethics Board (Protocol# 20200322-01 H) and Queen’s University Health Sciences & Affiliated Teaching Hospitals Research Ethics Board (Protocol# REH-773-20).

This study was conducted at The Ottawa Hospital (TOH), including the Civic and General campuses. TOH is a 900-bed tertiary care Health Sciences Network that is the regional referral center for trauma, vascular, neuro, thoracic and complex oncology surgery, and serves a catchment population of 1.2 million people. Both campuses are located in Ottawa, Canada.

Participants were recruited by emailing or mailing a recruitment poster (Supplemental Material 1) to older adults who were transitioning home after surgery. The email/mail requested that the recruitment poster be provided to their informal caregiver. If the informal caregiver was interested in participating, they contacted the lead researcher (EH), who explained the goals of the research and obtained informed verbal consent over the telephone. The participants were not known to the researchers. There is variability in the definition of an informal caregiver across research studies [3]. For this study, an informal caregiver was defined as someone known to the patient who provided some form of unpaid support and care. The patient and the informal caregiver mutually agreed that they were an informal caregiver during the postoperative transition in care. Caregiver participants were recruited within the year following the patient’s surgery. The patients were older adults (≥ 65 years) with a Clinical Frailty Scale score of ≥ 4 [15], and had an inpatient elective surgery.

### Data collection

Demographic information was collected using a telephone-based questionnaire. Telephone interviews were conducted using a semi-structured interview guide (Supplemental Material 2). The interview guide was informed by a continuum of care framework [16] that highlights the transition points for a hospitalization (admission to hospital, hospital stay and discharge planning, transition out of hospital) to help participants identify what was most important in supporting the postoperative transition in care. The interview guide was reviewed and revised by a patient partner (GK). All telephone interviews were conducted by EH. To mitigate the potential limitations of telephone interviews, the interviewer (EH), consistently checked-in with participants throughout the interview to ask how they were feeling about the discussion, if they had anything they would like to add or expand on, and were asked to clarify what they meant when they used certain language. During the consent discussion and throughout the interview, participants were reminded that they could pause or stop the interview at any time. Caregiver interviews ranged in time from 19 to 51 min. One interview (19 min) was stopped prematurely as the participant described feeling stressed thinking about the first few days at home after surgery and did not want to continue the interview at another time. Recruitment and data collection ended once information power [17] was achieved. This was evaluated by appraising the study aim, sample specificity, quality of the dialogue and analytic technique [17], while considering the recommended sample size (between 6 and 16 participants) when doing thematic analysis [18]. The authors (EH, JM) assessed these components and recruitment ended once the authors felt that there was quality and comprehensive data to support the objective of the research. EH took field notes during and after interviews, and participated in reflexive journalling, to document the meaning assigned to certain phrases, and to highlight emotions that came up for participants and the interviewer.

### Analysis

Interviews were audio-recorded, transcribed, reviewed for accuracy and then analyzed using reflexive thematic analysis [19] in an interpretive description tradition [12] to identify clinically relevant themes. A combination of coding on hardcopy transcripts and using Excel software was used for organizing data within codes, themes and subthemes. Rigor was maintained during analysis through an audit trail, documenting analytic field notes and engaging with the research team to encourage reflexivity [20].

Analysis followed the steps of reflexive thematic analysis: [19] (1) dataset familiarization; (2) data coding; (3) initial theme generation; (4) theme development and review; (5) theme refining, defining and naming; and (6) writing up. One study investigator (EH) independently coded the transcripts. Once codes were identified, a coding tree was developed (Supplemental Material 3). Two investigators (EH, JM) met to critically reflect upon the codes and initial themes, not to reach consensus but to develop a more reflexive and refined analysis [19]. The study was conducted and reported in alignment with the Consolidated Criteria for Reporting Qualitative Research checklist (COREQ) [21].

## Results

### Participants and characteristics

Eight participants consented to participate, and one withdrew from the study and therefore did not complete the interview. Demographic data for the 7 participants who completed the interview are presented in Table 1. While 2 of 7 informal caregivers did not live with the patient prior to surgery, they both moved in with the patient for a short time after surgery. There were 2 informal caregivers for the same patient (one was a spouse, and one was an adult child). Four patients had surgery before the COVID-19 pandemic was declared, two had surgery after the COVID-19 pandemic was declared, and one patient asked to withdraw from the study so no information on this patient is available. Surgery dates ranged between December 2019– March 2022. All of the interviews with the caregivers occurred within 3–9 months of surgery, and all occurred during the COVID-19 pandemic.

**Table 1:** Participant characteristics

Ages	39, 48, 62, 71, 76, 77, 82
Gender, n (%)	Man– 1 (14)
	Woman– 6 (86)
Relationship to patient, n (%)	Spouse– 4 (57)
	Adult Child– 3 (43)
Living with patient, n (%)	Yes − 5 (71)
	No − 2 (29)
Self-reported health status, n (%)	Poor − 0 (0)
	Fair − 3 (43)
	Good − 2 (29)
	Very Good − 1 (14)
	Excellent − 1 (14)
Education, n (%)	Some high school − 1 (14)
	Completed high school − 2 (29)
	Diploma/undergraduate degree − 4 (57)
Patient’s surgery type, n (%)	Gynecological– 2 (33)
	Thoracics– 2 (33)
	Urology– 1 (17)
	Otolaryngology– 1 (17)
Patient’s CFS score, n (%)	4 [vulnerable]– 3 (50) 5 [mildly frail]– 3 (50)
Time between surgery and interview, n (%)	3 months– 1 (17) 4 months– 1 (17) 7 months– 2 (33) 9 months– 2 (33)

### Themes

**Fig. 1:**
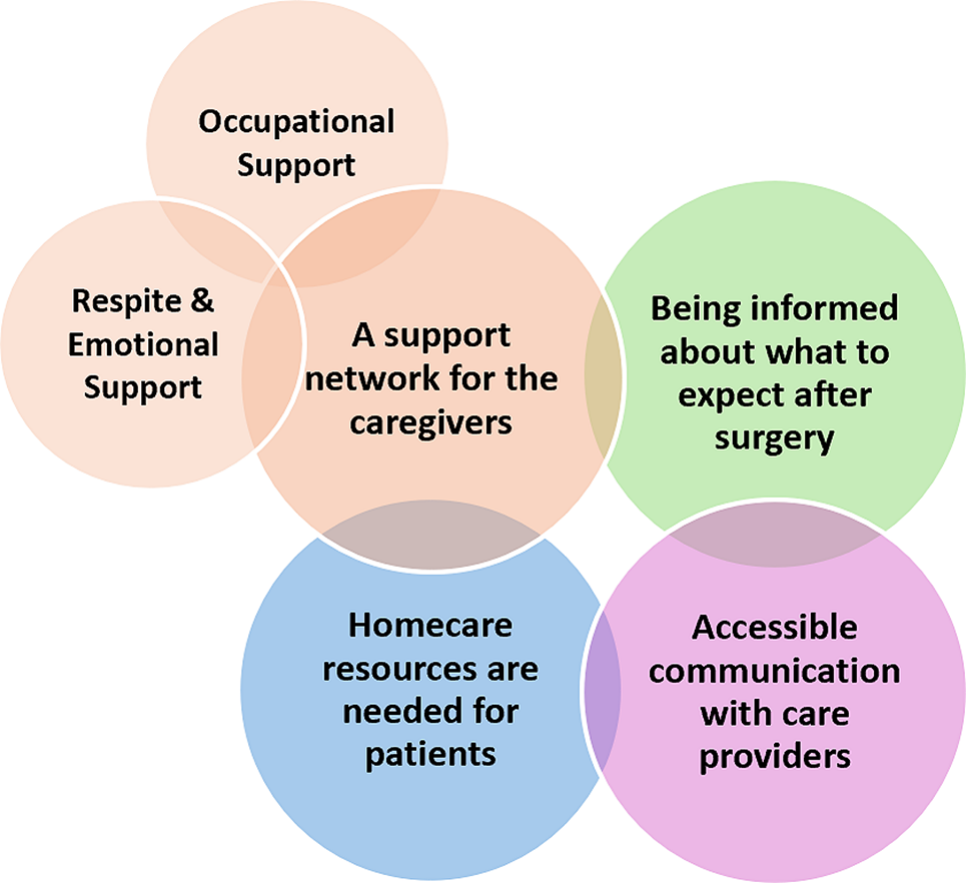
Themes and sub-themes. A visual of the 4 themes, including the theme that has 2 sub-themes

Four themes were constructed upon exploring what is important during a postoperative transition in care from the perspective of informal caregivers (1) being informed about what to expect after surgery; (2) accessible communication with care providers; (3) homecare resources are needed for the patient; and (4) a support network for the caregivers. Theme 4 included two sub-themes: (a) respite and emotional support and (b) occupational support. See Fig. 1.

#### Theme 1: Being informed about what to expect after surgery

Participants wanted to be informed about what the recovery trajectory might look like for the patient. They wanted to know how to monitor and manage postoperative symptoms and when to seek medical attention. One participant shared:

At least to know what the expectations are and to know that she’s progressing, more or less on a schedule that’s expected for someone her age, so that I know whether or not I need to be, contacting someone to say, you know, this is not looking right to me… because I don’t have a medical background. I don’t know what I don’t know so it would be good to know what to look for. C-005.

Another participant expressed wanting to know how to monitor for infections after surgery:

I think telling the caretakers what signs to look for, for infection and things that could go wrong, you know? So you know what to push the panic button for and what to expect and all that would be helpful. Even looking at the incision, did it look inflamed? I know my husband asked me a couple of times does it look, ah, it’s really sore here, does it look puffy and all red and all that? And it’s hard to tell because to you it looks terrible anyway, you know? Because it was a big, a big invasive, surgery. So, to me it always looked red and puffy and you know? C-004.

#### Theme 2: Accessible communication with care providers

Participants felt that they did not have access to a healthcare provider after surgery. One participant said how it would be essential to speak to a care provider who knows what the patient has been through and who can help answer questions:

I just find that there is no follow-up. No help from, um, you know after you’re out that hospital door it just seems that’s it… just to know that you can phone somebody. Not necessarily that you had to go into emergency. But you could just phone somebody. You know and ask a question. Somebody directly who answers, who knows what he’s been through. C-003.

Another participant said how they were unsure of who to contact and wanted a single point of contact to mitigate the feeling of experiencing so many moving pieces:

Well who do I speak to now? Well no, you have to call the nurse’s station. And then okay, nobody’s answering that phone. She hasn’t called me back. It’s been day one, I’ll try again. Ah, you know, day two… now I’ll try someone else. So there’s just so many pieces to the puzzle and it’s not always very clear who you’re supposed to talk to. I think that having someone assigned, not necessarily a doctor, but having someone be a single point of contact. You know, who I can call that would guide me… So having a single point of contact, someone assigned that we could feel that we didn’t have to chase for answers would be really helpful. C-005.

#### Theme 3: Homecare resources are needed for patients

Participants expressed a need for more community and homecare resources to help support the patients during their transition home after surgery. This included a need for nursing homecare and community resources to monitor how the patient is doing at home.

The need for support in the home was apparent when one participant said:

Well just, you know, looking after him, you know, his meals and his, you know, his bed and you’re looking after his bed and, and helping him get up when - if he needed help and that. It’s just never easy. He’s 6 foot 4. I’m 5 foot 2. C-003.

Another participant described how there are not enough resources in the community to provide personal support services, which they expressed is particularly important when the informal caregiver is also an older adult and may not be in the best health:

Um, you know in terms of personal support workers. There’s so many people who come out of hospital, um, where they would qualify for a certain amount of personal support services, whether it be in their situation it’s daily or twice a day or, um, maybe in a situation every other day and, and there are a lot of times when that it is just not happening because here’s not enough resources in the community. It’s fine where there’s family supports that can fill in family, friend neighbour, but there’s a lot of times where people don’t have children and they’re in their 80s and the caregiver, primary caregiver in the home would not rate their health as excellent but maybe moderate. C-002.

Another participant said that she could not find any community resources to support her mother after surgery who lived in a different town:

I did do a bit of searching on the internet and friends did as well, to try and find out what kind of resources, if any, in a smaller community, might be available to her, but we didn’t find anything… it would have been very helpful for both of us in the sense that she really, in some ways, was anxious to get home, which I could understand. She wanted to be, amongst her own stuff… We all do. She wanted to not feel like a burden. But there’s nobody there for you. What happens if you fall? So you know, in some senses I may have kept her longer than she wanted to stay because I didn’t feel that she would have the support that she needed when she went home. C-005.

#### Theme 4: A support network for the caregivers

Participants highlighted the value and need for a support network for themselves during the postoperative transition in care. While the support network might have looked different for adult child caregivers versus spouse caregivers, the premise was that informal caregivers require a network to provide the support they need in their context. This included respite and emotional support as well as occupational support.

#### Respite and emotional support (sub-theme A)

One participant described the emotional toll of caregiving and the desire for respite:

Um, it’s a stressful, trying time for the caretaker. I think if you’re not emotionally involved like a PSW or a nurse that comes to the house, it’s totally different because you can go home. But when it’s the family member, it’s different. You know? It’s, um, 24/7 it’s you’re emotionally involved, right? So it’s not a job where you get paid for eight hours and then forget about it, you know? The respite would be nice so a person can go out for a couple of hours and do whatever they wanted, you know? C-004.

Another participant explained how it would have been helpful to have someone to talk to who understood what they were going through:

If we’d have had that one point of contact, you know that person might have also presented a reminder to say ‘no matter how much you want to do this. This is going to be stressful on you too. You need to understand that and cut yourself some slack. Um, and make sure that you carve out a bit of time for yourself’… Ah, so that you don’t feel overwhelmed, guilty… because I think understanding that what you’re going through is normal, helps release some of the pressure… So that you go, okay, you know what? I don’t have to be perfect. And what I’m going through was quite normal. Everybody goes through it. Just breathe. C-005.

#### Occupational support (sub-theme B)

One participant shared the challenges of working while being an informal caregiver:

And then of course, I’m having to drop everything that, you know, in my workday because I’m still working… I’m going to my boss, “Okay, like I’m offline for the next hour or two because I need to find out what’s going on with my mother.”… not that I’m all that concerned about it, but some people could be. It’s lost wages… and then of course, you’re in a mental mindset that, you know, well I’m not going back to work today because I deal with logic that’s complex and my brain just isn’t in a good headspace… So, it affects me on a number of levels. C-005.

Another participant explained how valuable their support system was regarding work:

It was definitely trying at times, but again, I have a great support system. So if I wasn’t able to take work off for whatever reason, then my husband was able to or my mother-in-law… I could only imagine the people that don’t have that support system. It would be 10 times worse for them… [my Mom] lost her license… Because she lost that independence. So she has no choice but to rely on all these other people. C-006.

## Discussion

This qualitative interpretive description study sought to explore and describe what was important to informal caregivers during a postoperative transition in care for older adults with frailty. Four themes were constructed, including (1) being informed about what to expect after surgery, (2) accessible communication with care providers, (3) homecare resources are needed for patients and (4) a support network is needed for caregivers. The fourth theme included 2 sub-themes: (a) respite and emotional support and (b) occupational support. The findings from this study of informal caregivers should be considered by future informal caregivers, clinicians, researchers and policy-makers when preparing for the role of a caregiver, as well as in developing and evaluating postoperative transitional care interventions and policies.

Caregiver participants wanted to be informed about what to expect after surgery, especially the postoperative recovery trajectory for their loved one. Some evidence is available to inform communications with caregivers that could address this need. For example, research suggests that 1 in 5 older adults with frailty who are having surgery experience worsening patient-reported disability in the early months after surgery, but that 9 in 10 improve by the one-year mark after surgery [22]. However, further research is still required to inform other dimensions of patient-centered recovery trajectories, including the longitudinal experience of informal caregivers. For example, participants in the current study wanted to know if their loved one was progressing in alignment with expected, positive recovery given patient age and type of surgery. Providing informal caregivers with anticipated timelines for achieving postoperative recovery milestones could help them to feel more confident in monitoring the recovery and progress of their loved one, and with knowing when to contact a healthcare provider with concerns. One qualitative study uncovered that for caregivers, having a disease trajectory would enhance their capacity to provide care by managing their expectations, informing what care interventions to pursue and might ultimately improve their own well-being as a caregiver [23]. As the health of a loved one declines, the magnitude of informal caregiving required increases [24]. Therefore, information about recovery would support logistical and financial planning for the postoperative phase (i.e., time off work, homecare, convalescence). Participants in the current study expressed uncertainty for how to support their loved one when they were feeling slowed up, were experiencing pain, or were struggling emotionally. Importantly, healthcare providers play an essential role in preparing informal caregivers for their role [25]. The preoperative period should incorporate communication of recovery trajectories, education and support for informal caregivers.

Participants also wanted to be informed about potential risks and how to monitor symptoms at home. Aligned with these findings, a prior scoping review highlighted that personalized risk communication before surgery has the potential to support the shared-decision making process and to allow for individualized care planning after surgery [26]. However, there is a need for evidence informing how similar information can be communicated with informal caregivers. Explicitly supporting caregivers in monitoring postoperative symptoms could also empower informal caregivers to monitor and triage symptoms accordingly, especially if a connection to clinical centers can be maintained. This could also support accessible communication with healthcare providers after surgery, another need identified by participants. This is in line with previous research that has highlighted the challenges that informal caregivers face with not knowing who to contact and difficulties with trying to navigate the healthcare system [23] Caregivers have expressed a desire for leveraging technology to support access to information and support with providing care [23]. One such example is remote automated monitoring (RAM), a virtual technique where biophysical variables and patient-reported symptoms can be captured using technology and observed by clinicians [27]. One qualitative study found that in the context of patients with kidney disease, patients and caregivers believed that RAM increased their knowledge of the disease, fostered clinician accountability and enhanced access to treatment and efficient care; however, patients and caregivers voiced concern that RAM should not replace human connection and face-to-face contact with clinicians when needed [28]. Research is needed to examine RAM in the context of postoperative transitions in care for older adults with frailty, including from the perspective of patients and informal caregivers. It is important to consider cost and the preferences and needs of informal caregivers, their comfort level, and their access to the internet and technology [29].

Participants voiced a need for more homecare support and services for patients recovering at home after surgery. While there was a need for more health homecare (nursing care), participants most often described requiring more supportive care (bathing, dressing, etc.,), particularly from informal caregivers who were older spouses. This is in line with the unmet care needs in the landscape of homecare in Canada, where there are more unmet needs for support services (bathing, meal preparation, housekeeping, transportation) than health homecare services (nursing care, physiotherapy, nutritional counselling) [30, 31]. An adult child caregiver also conveyed that no community or homecare services were available for their parent who lived in a small town. Older adults who live in small or rural towns have been found to receive less homecare than older adults living in urban areas [32]. More accessible and publicly-available homecare has been identified as a priority by patients and caregivers [33] yet funding restrictions and strict eligibility criteria continue to hinder the availability and access to homecare for older adults [31]. Future homecare priority settings should engage informal caregivers who provide care and support to older adults with frailty.

Further, participants relied on a support network during the postoperative transition in care. Participants turned to their support network for respite and emotional support. Participants described wanting to have respite from caregiving. However, respite care has been described as “inflexible” and isn’t available to many informal caregivers, due to strict eligibility criteria [34]. Further, there can be hesitation for informal caregivers to accept respite care. This can be for a variety of reasons including caregiver and patient preferences that the informal caregiver is the individual who knows the patient best and should be the one providing support [35]. It is important to expand access to respite care and to investigate ways to provide respite to informal caregivers in a way that provides comfort to both the informal caregiver and patient. Additionally, participants in the current study shared that they wanted someone to talk to who understood their experience. A systematic review of training for informal caregivers of older adults suggests that support interventions (i.e., telephone-based psychotherapy, case management, interdisciplinary support, e-learning platform) have the potential to decrease stress and improve quality of life among caregivers [36]. However, among the 24 included articles in the review, none of the articles included informal caregivers to older adults having surgery [36]. It would be valuable to engage informal caregivers in the design and development of future support interventions for informal caregivers in the context of perioperative medicine.

Participants also relied on their support network for occupational support. This is in line with a systematic literature review of the preferences of informal caregivers which emphasized the need for respite and work-life balance, and the value of sharing the responsibility of care to match these needs [37]. For working caregivers, professional support is one of the most challenging aspects to achieve while providing informal care [37]. This is important to consider as 1 in 4 Canadians of working age are informal caregivers and are at risk of job insecurity as a result [34, 38, 39]. Approximately 1 in 20 employed informal caregivers either leave the workforce by retiring, quitting or being terminated as a result of the challenges with balancing work and caregiving [34, 38]. While informal caregivers receive 2 weeks of paid leave from work to provide caregiving support in the Netherlands [9], this is not common practice in other parts of Europe or North America, but perhaps should be considered by policy-makers. Alternatively, flexibility in working hours could support the occupational needs of informal caregivers [40]. As the Canadian Compassionate Care Benefit (CCB) is only available to informal caregivers who are caring for someone who is at end-of-life [41], there remains a need to provide support to employed informal caregivers to other patient populations, including older adults recovering from major surgery. The Canadian Centre for Caregiving Excellence has stated that existing financial support for informal caregivers is insufficient and inaccessible, and that there is an urgent need to address the financial burden and out-of-pocket costs that informal caregivers endure [34]. Each year, informal caregivers provide the same amount of care as 2.8 million full-time paid care providers [34]. Despite being described as one of the main support structures in society [34], and experiencing many costs associated with caregiving, informal caregivers face the very real threat of unemployment and financial stress amidst trying to provide care to their loved one. Due to the lengthy wait-lists for long-term care [42] and unavailability of homecare [30], informal caregivers are essential. It certainly brings into question, if not informal caregivers, then who? Solutions to support informal caregivers, especially employed informal caregivers, is urgently required [34].

Transitions in care present challenges for informal caregivers of older adults with frailty, who play an important role in successful transitions. This study highlighted what is important to informal caregivers during a postoperative transition in care that should be considered by relevant groups to facilitate successful transitions in care after surgery. More research is required to provide informal caregivers with information regarding recovery trajectories for older adults with frailty having surgery and should be communicated clearly before surgery to informal caregivers to help with planning. Informal caregivers should be provided with the knowledge and resources for monitoring postoperative symptoms and should be provided with clear contact information and access to a healthcare provider to address any questions or concerns regarding the recovery process. More publicly-funded and accessible homecare services are required, most notably home support services. Informal caregivers require respite and emotional support, and future research is required to understand ways in which this can be provided in a way that matches the needs and preferences of the informal caregiver and patient. Finally, there is a need to provide occupational support, including paid time-off, to help support informal caregivers who are also working.

## Strengths & limitations

The strengths and limitations of this study should be considered. This study focused on the experiences of informal caregivers to a population that is often under-represented in research (older adults living with frailty). However, many surgical patients without frailty and their caregivers may have similar views on what is important to them during a postoperative transition in care. While this study included a varied sample of informal caregivers, a purposive sampling was not possible due to the limitations of the recruitment strategy, whereby caregivers contacted the researcher if they were interested in participating. Further, this research was conducted during the COVID-19 pandemic, when the strain on the healthcare system, patients and caregivers was exacerbated. However, the participants in the study did not commonly describe pandemic-related challenges with their experiences. While participants described being confident in remembering their experience during the postoperative transition in care, some of the caregiver interviews took place several months following surgery and their experience, which may have affected their recall. As this study solely focused on the postoperative transition in care following elective surgery, there is a need for future research to explore this in the context of emergency surgeries. Further, participants were recruited from within the same city, which may limit the transferability of our findings. Future work should consider the experiences of those living in other areas.

## Conclusion

Informal caregivers play a significant role during postoperative transitions in care for older adults with frailty. Clinicians, researchers, homecare service agencies and policy-makers should consider the results of this study, which highlight the aspects of transitions in care that are important to informal caregivers, when conducting new research and developing policies to support transitions home after surgery for older adults with frailty.

### Electronic supplementary material

Below is the link to the electronic supplementary material.

Supplementary Material 1

Supplementary Material 2

Supplementary Material 3

## Data Availability

All data generated or analysed during this study are included in this published article [and its supplementary information files]. Any additional data are available from the corresponding author on reasonable request.

## Abbreviations

ADL: Activities of Daily Living
CCB: Canadian Compassionate Care Benefit
TOH: The Ottawa Hospital
RAM: Remote Automated Monitoring
