# Supplementary material for: “It’s a stressful, trying time for the caretaker”: an interpretive description qualitative study of postoperative transitions in care for older adults with frailty from the perspectives of informal caregivers
Source: BMC Geriatr. 2024 Mar 11;24:246. doi: 10.1186/s12877-024-04826-4 (PMC10929104; doi:10.1186/s12877-024-04826-4)
Supplement: Supplementary file 1 — Supplementary Material 1 [file 12877_2024_4826_MOESM1_ESM.docx]

**Postoperative transitions in care for older adults with frailty: understanding patient and caregiver priorities**

***Protocol #20200322-01H***

**Were you a caregiver to an adult (aged 65 and older) who had major surgery at The Ottawa Hospital within the last year?**

- We are doing a research study to better understand YOUR experience as a caregiver to an older adult during their transition home after surgery.
- We hope to draw on your experience to improve the transition home after surgery based on the priorities that are important to patients and caregivers.
- Your participation would include a brief questionnaire and a telephone interview.

Please call Emily Hladkowicz at 613-292-6714 or email her at [emhladkowicz@toh.ca](mailto:emhladkowicz@toh.ca) if you are interested in participating!

This study is being done as a part of Emily’s PhD thesis through Queen’s University and The Ottawa Hospital Research Institute.

**P**
