# Supplementary material for: “It’s a stressful, trying time for the caretaker”: an interpretive description qualitative study of postoperative transitions in care for older adults with frailty from the perspectives of informal caregivers
Source: BMC Geriatr. 2024 Mar 11;24:246. doi: 10.1186/s12877-024-04826-4 (PMC10929104; doi:10.1186/s12877-024-04826-4)
Supplement: Supplementary file 2 — Supplementary Material 2 [file 12877_2024_4826_MOESM2_ESM.docx]

**Interview Guide**

*Hello,*

*Thank you for agreeing to participate in this telephone interview. I am interested in speaking with you today because an older adult that you provided care for recently had major, elective non-cardiac surgery. As a result, they would have also experienced a transition from hospital to home, which is what we call a “postoperative transition in care”. As a caregiver, you would have likely been involved in aspects of this process. A transition in care is defined as, ‘a set of actions designed to ensure the coordination and continuity of healthcare as patients transfer between different locations”. Most of the time, a transition in care is described as successful when a patient is discharged from hospital and does not get readmitted back to hospital. The purpose of this interview is to understand what was important during the postoperative transition in care process for you and how you would describe a successful postoperative transition in care.*

1. Can you please start by describing your overall experience of being a caregiver to someone during their transition home after surgery?
2. What happened before surgery that helped their transition home?

- What did you find most important?
- What would have been important?

1. What happened in the hospital after their surgery that helped you, as a caregiver, prepare for their transition home?

- What was most important to you during their hospitalization to prepare you for their transition home? (i.e. people, resources, etc.)
- Was there anything missing/things that could have been improved that would have been helpful?

1. What was the discharge planning process like, and what did you find most important to you?

- Were you involved in the discharge planning? Would this be important to you?
- Were discharge instructions provided and explained to you? Were they clear?
- Was here anything missing / things that could have been improved that would have been helpful and important to you?

1. Can you describe the first few days that the patient was at home.

- What was it like for you? What type of support did you provide?
- What was helpful for the transition home?
- What was important to you during their first few days at home?
- What was challenging about the transition home?
- What could have been improved?

1. Can you please explain any other services you required/sought out once he patient was discharged home?

- Did they see their family doctor for follow-up?
- Were there home-care services in place?
- What was important for their transition once they were home?
- Was there anything missing once home that would have been be important?

1. If you were to be a caregiver again for another older adult requiring a postoperative transition in care (having surgery again and going from hospital to home), what would be important to you during the transition as a caregiver (i.e., clear discharge education, early follow-up with GP, caregiver involvement, etc.)?
2. Would you say that your loved one/family member/friend’s postoperative transition in care was successful? If yes, what made it successful?
3. If no, what would make a postoperative transition in care successful in your mind?
4. Is there anything else you would like to add to help me understand what was/would be important to you during a transition home after surgery from the perspective of a caregiver?
5. Is there anything else you would like to add to help me understand what it would mean, and how I would know, that you have had been involved in a successful transition home after surgery?

Thank you so much for your time.
