# Supplementary material for: “It’s a stressful, trying time for the caretaker”: an interpretive description qualitative study of postoperative transitions in care for older adults with frailty from the perspectives of informal caregivers
Source: BMC Geriatr. 2024 Mar 11;24:246. doi: 10.1186/s12877-024-04826-4 (PMC10929104; doi:10.1186/s12877-024-04826-4)
Supplement: Supplementary file 3 — Supplementary Material 3 [file 12877_2024_4826_MOESM3_ESM.docx]

Caregiver Decision Tree

| **Theme** | **Code** | **Quotes** |
| --- | --- | --- |
| Being informed about what to expect after surgery | Apprehensive about what to expect with surgery | *Um, for the type of surgery my mom had, um, and my mom not having extensive surgery before, ah, I was certainly apprehensive as to how she would respond, ah, in terms of, ah, coming home with her—regards to her mobility and pain control.* |
|  | Communication about what to expect so you can be prepared at home | *Um, I think as long as you’re completely transparent in what the possibilities could be so you can prepare for the worst… The whole communication, I think that is key, is just the communication being told what to ex—exactly what to expect, so you can have the proper things in place at home.* |
|  | What to expect in terms of symptom management | *I think telling the caretakers what signs to look for, for infection and things that could go wrong, you know? So you know what to push the panic button for and what to expect and all that would be helpful. Even looking at the incision, did it look inflamed? I know my husband asked me a couple of times does it look, ah, it’s really sore here, does it look puffy and all red and all that? And it’s hard to tell because to you it looks terrible anyway, you know? Because it was a big, a big invasive, surgery. So, to me it always looked red and puffy and you know?* |
| Accessible communication with care providers | To have a phone number to call instead of going to the hospital | *But, but, you know, like I said, he did have, um, in his catheter, he had blood clots and we weren’t sure whether that was common or whatever, you know? And so we were a little concerned about that. But, you know, and you wait and you—should you phone somebody or, you don’t who phone. It’s not like something that you would, um, ah, rush to emergency for… You know, so you knew. But you didn’t have a phone number. That’s the main thing, I think. You didn’t have a contact person…And I think that was the biggest.* |
|  | Communication and continuity of care with the healthcare system | Um, yeah. It would have been nice to, um, to give us both a sense of, um, that, that she was being taken care of … Um, and, ah, that, that, you know, somebody in the system cared … Um, so, you know, that, that would have, I think, relieved some stress on us rather than just, you know, ah, kind of knowing okay, how are you feeling? Everything is good. You know, okay. Um, and, and having to rely on our own judgement. |
| Homecare resources are needed for patients | Nursing homecare needs to be made more available | Um, you know certainly she might have been able to, you know, pay to have a nurse come in. Um, but I didn’t—I did not come across any, um, thing that was like from the Ontario government or something that would be, ah, that was made available to her ...it would have been nice if there, ah, if a nurse could have stopped by. |
|  | Personal support homecare is needed in the community | *Um, you know in terms of personal support workers. There’s so many people who come out of hospital, um, where they would qualify for a certain amount of personal support services, whether it be in their situation it’s daily or twice a day or, um, maybe in a situation every other day and, and there are a lot of times when that it is just not happening because here’s not enough resources in the community.* |
|  |  | I think on the way after, the way home, you know, that—that to me, because you’re leaving it all up to the hospital before… And then it’s after that you really need some help. Support, not, not help necessarily. But… support. |
| A support network for the caregivers – respite and emotional support | Getting away is important to take time for yourself | And like I said, I went out for a drive and, and I was at times when, um, I, I did go over and sit like when the weather was nice, sit on a, my veranda or outside with friends, you know? And, um, so that all helped just to get away, you know? And then you have to remember that they’re in pain and they’re, ah, and especially when the cancer came back you have a fear of him dying, too. |
|  | Feeling cared about and having emotional connection | I guess I could have called the minister. But then he called and checked in on us and he made sure we were okay, too … Well it’s just somebody that cared and, you know, was thinking of us. |
| A support network for the caregivers – occupational support | Family helps to provide support because of the demands of being a working caregiver | *I think it was just so not everything was thrown onto my plate, right? Like we’re, we’re busy. We have three kids—well, 11, 12 and 14. So at least they’re old enough. I couldn’t imagine if they were still two, three and five ...But the fact that their—they were a little older and they could help out a little bit more and plus I work full time and then I had to work from home because schools were shut down. So I think it just made my life a little easier having that support system. So it just wasn't all put on me.* |
|  | Challenges of working while being an informal caregiver – time off work or more available homecare services | Well that’s it, like I’m thinking if my mom had another kind of surgery where she couldn’t walk. Well, that would be huge because I would have no choice but to take time off, because she wouldn’t be able to get to the bathroom ...You know what I mean? So those are all things that, that people take for granted, really. Um, ah—so yeah, I don’t know. Like initially about having someone come to the house to, to help take care for her when I went back to work ... Yeah. Now the only other thing with that is that can be pretty expensive ... So like maybe having more programs and stuff that make it more feasible for people ... I guess that would be the only thing that I would challenge, is make it easier for people to obtain the services out there because I was just fortunate that I didn’t need them, but that’s this time. Who knows if there is going to be a next time that’s going to be even worse? Now and I don’t know, maybe they didn’t’ provide some of this information to me because they knew that my mom was living with me. |
